# Rumen and Serum Metabolomes in Response to Endophyte-Infected Tall Fescue Seed and Isoflavone Supplementation in Beef Steers

**DOI:** 10.3390/toxins12120744

**Published:** 2020-11-26

**Authors:** Taylor B. Ault-Seay, Emily A. Melchior-Tiffany, Brooke A. Clemmons, Juan F. Cordero, Gary E. Bates, Michael D. Flythe, James L. Klotz, Huihua Ji, Jack P. Goodman, Kyle J. McLean, Phillip R. Myer

**Affiliations:** 1 Department of Animal Science, University of Tennessee, Knoxville, TN 37996, USA; tault1@vols.utk.edu (T.B.A.-S.); eam@nmsu.edu (E.A.M.-T.); Brooke.Clemmons@tamuc.edu (B.A.C.); Juan.Cordero@utk.edu (J.F.C.); kmclea10@utk.edu (K.J.M.); 2 Department of Plant Sciences, University of Tennessee, Knoxville, TN 37996, USA; gbates@utk.edu; 3 USDA-ARS, Forage-Animal Production Research Unit, Lexington, KY 40546, USA; michael.flythe@ars.usda.gov (M.D.F.); James.Klotz@ars.usda.gov (J.L.K.); 4 Kentucky Tobacco Research and Development Center, University of Kentucky, Lexington, KY 40546, USA; hji4@email.uky.edu; 5 Department of Plant and Soil Sciences, University of Kentucky, Lexington, KY 40546, USA; jpgood2@email.uky.edu

**Keywords:** beef cattle, endophyte, ergot alkaloid, fescue toxicosis, isoflavone, metabolites

## Abstract

Fescue toxicosis impacts beef cattle production via reductions in weight gain and muscle development. Isoflavone supplementation has displayed potential for mitigating these effects. The objective of the current study was to evaluate isoflavone supplementation with fescue seed consumption on rumen and serum metabolomes. Angus steers (*n* = 36) were allocated randomly in a 2 × 2 factorial arrangement of treatments including endophyte-infected (E+) or endophyte-free (E−) tall fescue seed, with (P+) or without (P−) isoflavones. Steers were provided a basal diet with fescue seed for 21 days, while isoflavones were orally administered daily. Following the trial, blood and rumen fluid were collected for metabolite analysis. Metabolites were extracted and then analyzed by UPLC-MS. The MAVEN program was implemented to identify metabolites for MetaboAnalyst 4.0 and SAS 9.4 statistical analysis. Seven differentially abundant metabolites were identified in serum by isoflavone treatment, and eleven metabolites in the rumen due to seed type (*p* < 0.05). Pathways affected by treatments were related to amino acid and nucleic acid metabolism in both rumen fluid and serum (*p* < 0.05). Therefore, metabolism was altered by fescue seed in the rumen; however, isoflavones altered metabolism systemically to potentially mitigate detrimental effects of seed and improve animal performance.

## 1. Introduction

Tall fescue is the major forage used to feed cattle in pasture-based systems of the southeast and covers approximately 14 million hectares across the United States [1]. The advantage of tall fescue is hardiness of the plants attributed to the presence of a fungal endophyte (*Epichloë coenophialum*, formerly known as *Neotyphodium coenophialum* and *Acremonium coenophialum*) living in a mutualistic relationship with the plant [2]. However, the endophyte produces ergot alkaloids that are toxic to animals that consume them for an extended period of time [3]. Ergot alkaloids are able to bind biogenic amine receptors on blood vessels, resulting in vasoconstriction throughout the body [4,5,6]. This results in a condition known as fescue toxicosis, which is commonly observed by the animal’s inability to thermoregulate [7], poor reproductive performance [1], and reduced average daily gain [8], significantly reducing overall animal performance. Therefore, researchers are tasked with identifying management methods and therapeutics to alleviate these consequences to cattle producers.

Pasture management methods have been evaluated for reducing the impact of fescue toxicosis in cattle. Inter-seeding of legumes, such as red clover, to mitigate the effects of fescue toxicosis has proved beneficial in cattle grazing endophyte-infected tall fescue [9]. Recent research has found phytoestrogenic compounds, known as isoflavones, present in red clover may be responsible for reducing the effects of fescue toxicosis. Isoflavones act as an agonist on the β-adrenergic receptors present on blood vessels to promote vasodilation [10], reversing the effects of ergot alkaloid induced vasoconstriction. Additionally, isoflavones act as a natural antibiotic selective against hyper-ammonia-producing bacteria (HAB) and some cellulolytic and amylolytic bacteria [11,12]. The reduction of ammonia levels as a result of less HAB in the rumen allows more amino acids to be absorbed and used by the ruminant, while altered celluloytic and amylolytic bacteria can influence the production of volatile fatty acids for energy. Therefore, the increase in blood flow and altered rumen fermentation may improve nutrient delivery and utilization for host metabolic processes contributing to animal growth.

The objective of the present study is to evaluate the effect of isoflavone supplementation with tall fescue seed consumption on beef steer’s rumen and serum metabolomes. Ruminal and circulating metabolites may provide insights into altered bacterial and host metabolic functions that improve steer performance on endophyte infected tall fescue with the administration of isoflavones.

## 2. Results

### 2.1. Global Rumen Fluid and Serum Metabolome Comparison

**Figure 1:**
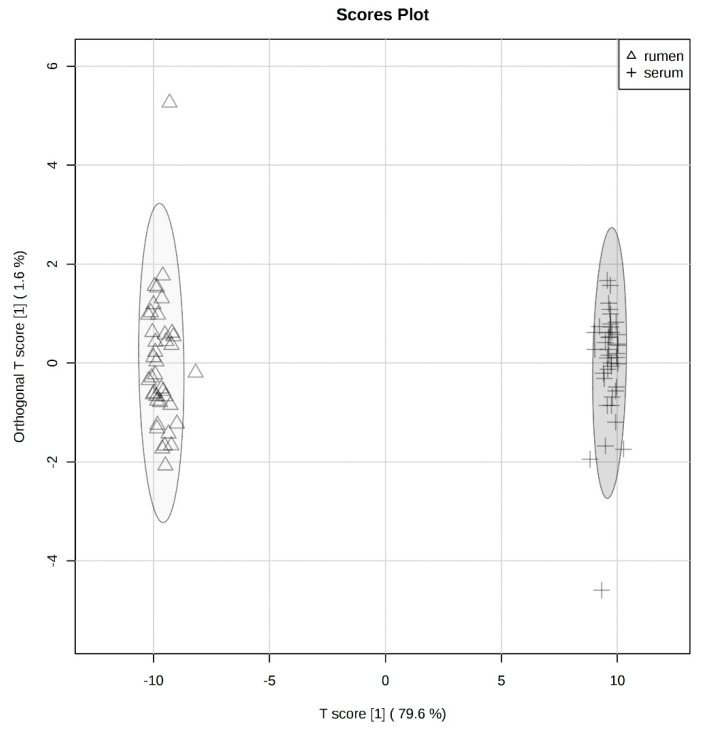
Orthogonal partial least squares discriminant analysis (O-PLS-DA) visualizing separation of rumen fluid (triangle) and serum (plus-sign) metabolomes. Ellipse represents a 95% confidence interval.

**Figure 2:**
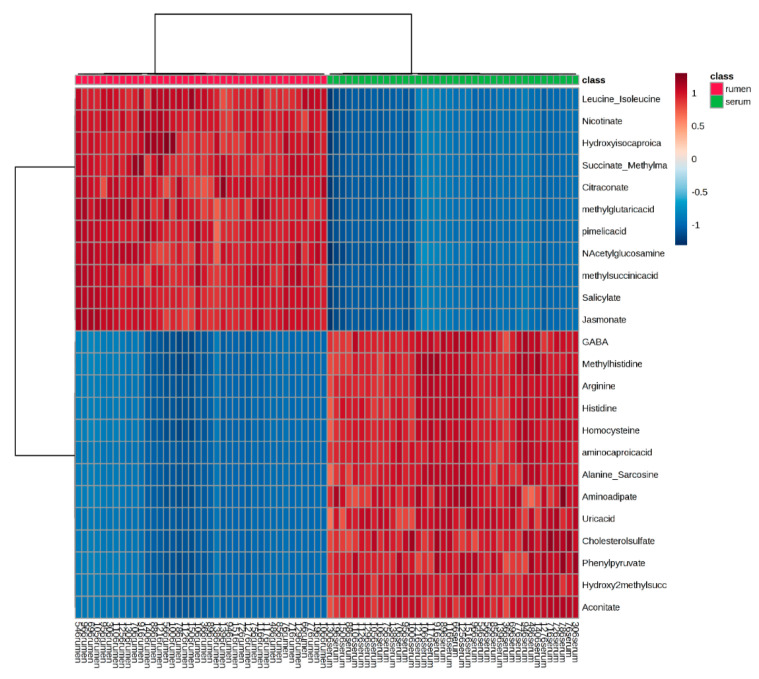
Heatmap of top 25 metabolites of rumen fluid and serum metabolomes by individual steers. Rumen fluid is represented by the red square at the top of the heatmap and serum metabolites are represented by the green squares.

An orthogonal partial least squares discriminant analysis (O-PLS-DA) was used to depict the relationship between the global rumen and serum metabolomes, which illustrated distinct separation between the two metabolomes (Figure 1). A heatmap was also used to visualize the top 25 rumen fluid and serum metabolites by individual steer (Figure 2). The heatmap supports that there is very little similarity between the overall ruminal and circulating metabolites.

### 2.2. Rumen Fluid Metabolome

**Figure 3:**
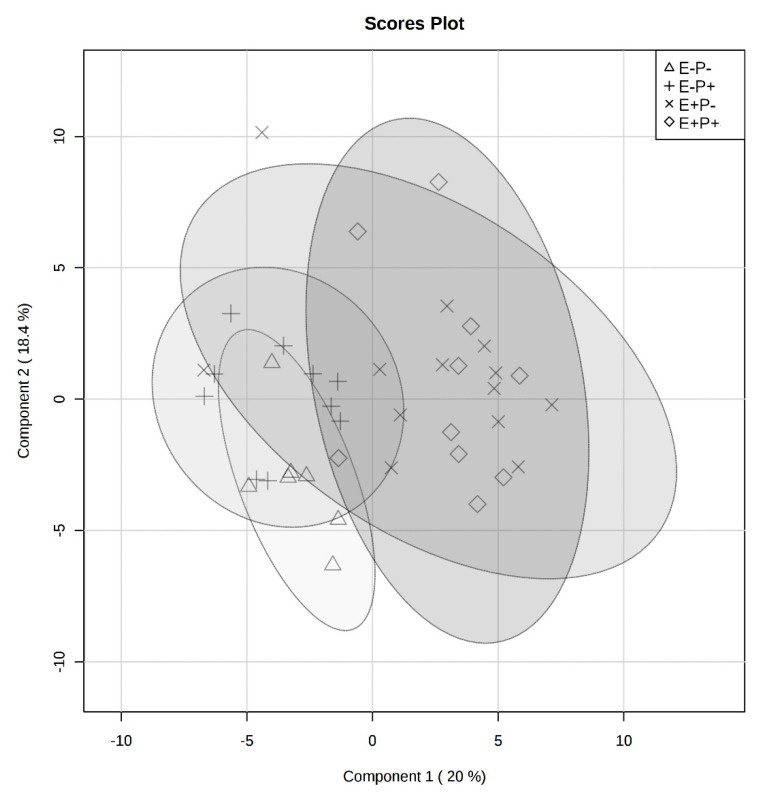
Partial least squares discriminant analysis (PLS-DA) visualizing differences in rumen fluid metabolomes between endophyte-free seed without isoflavones (triangle), endophyte-free with isoflavones (plus-sign), endophyte-infected without isoflavones (multiplication-sign), and endophyte-infected with isoflavones (diamond) treatment groups. Ellipse represents a 95% confidence interval.

**Figure 4:**
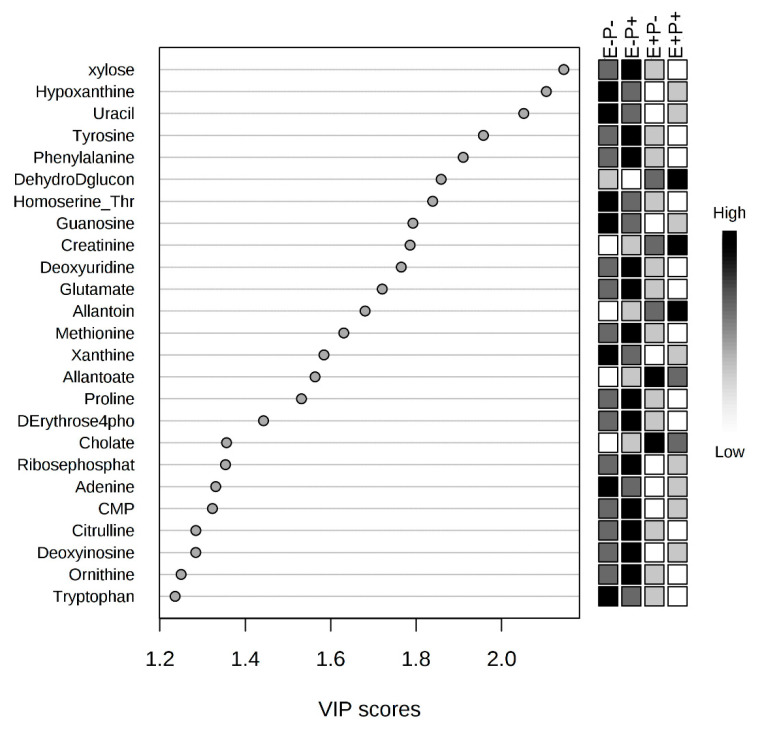
Variable importance in the projection (VIP) plot indicates xylose to have the greatest influence on the differences in rumen fluid metabolomes between all treatment groups.

All identified rumen fluid metabolites are presented in Supplementary File 1 with means and standard errors of the mean by treatment combination group. To visualize the effect between steers of the E+P+ and E−P− groups on the rumen fluid metabolome, a partial least squares discriminant analysis (PLS-DA) was created and a significant distinct overlap among seed type was noted (Figure 3). Correlation analyses were performed to analyze the correlation of individual rumen metabolites with the treatment combination groups, and variable importance in the projection (VIP) scores were generated to determine the metabolites that contributed to variation in rumen fluid metabolomes among treatment combination groups. Xylose was negatively correlated with the treatments (r = −0.57) and had one of the greatest impacts on metabolome differences among all treatments (*p* = 0.01) (Figure 4, Table 1). Individual metabolite and metabolic pathway analyses were not significantly impacted by the interaction of seed type and isoflavone treatments (*p* > 0.05).

**Figure 5:**
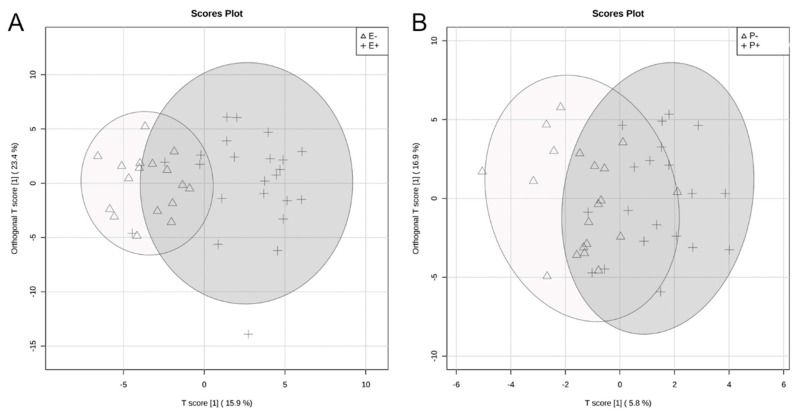
Orthogonal partial least squares discriminant analyses (O-PLS-DA) visualizing differences in rumen fluid metabolomes by seed type (**A**) and isoflavone (**B**) treatments. For seed type (**A**), endophyte-free (E−) steers are represented by a triangle and endophyte-infected (E+) steers by a plus-sign. For isoflavone treatments (**B**), steers receiving isoflavones (P+) are represented by a plus-sign and without isoflavones (P−) by a triangle. Ellipse represents a 95% confidence interval.

**Figure 6:**
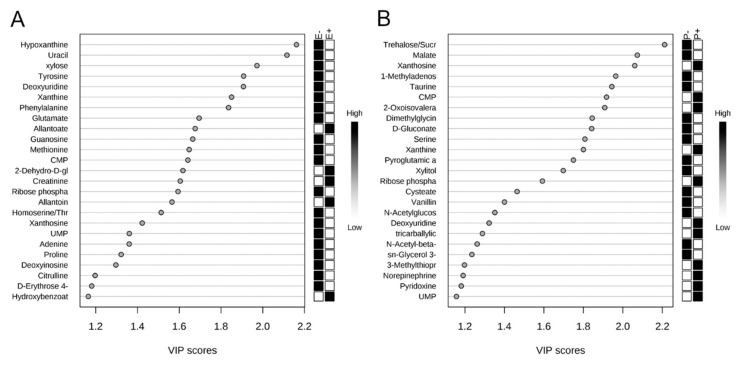
Variable importance in the projection (VIP) plots indicate hypoxanthine to have the greatest influence on the differences in rumen fluid metabolomes between endophyte-free (E−) and endophyte-infected (E+) seed treatment groups (**A**), and trehalose sucrose to have the greatest influence between isoflavone treated (P+) and control (P−) groups (**B**).

The rumen metabolome was analyzed by the main effects of seed type and isoflavone treatment. In order to visualize the difference in rumen fluid metabolomes, an O-PLS-DA was generated for the main effects of seed type (Figure 5A) and isoflavone treatment (Figure 5B). For seed type and isoflavone treatment, partial separation was observed between endophyte-infected and endophyte-free seed (Figure 5A). Additionally, partial separation was observed between steers receiving isoflavones and those that did not receive isoflavones (Figure 5B). Correlation analysis indicated hypoxanthine was negatively correlated (r = −0.56) and determined by VIP analysis to have a significant impact on the rumen fluid metabolome differences between endophyte-infected and endophyte-free seed treatments (*p* = 0.01; Figure 6A; Table 1). For isoflavone treatments, trehalose/sucrose was positively correlated (r = 0.06), but had no impact on the rumen fluid metabolome differences (Figure 6B). Metabolites that differed by seed type are presented in Table 1. Eleven metabolites differed significantly as a result of endophyte-infected versus endophyte-free treatments (*p* < 0.05, Table 1). No individual metabolite differences were observed in the rumen fluid as a result of isoflavone treatment. Metabolic pathways that differed significantly by seed type or isoflavone treatment are presented in Table 2. Twenty metabolic pathways were affected by seed type, but only two pathways were affected by isoflavones (*p* < 0.05).

### 2.3. Serum Metabolome

**Figure 7:**
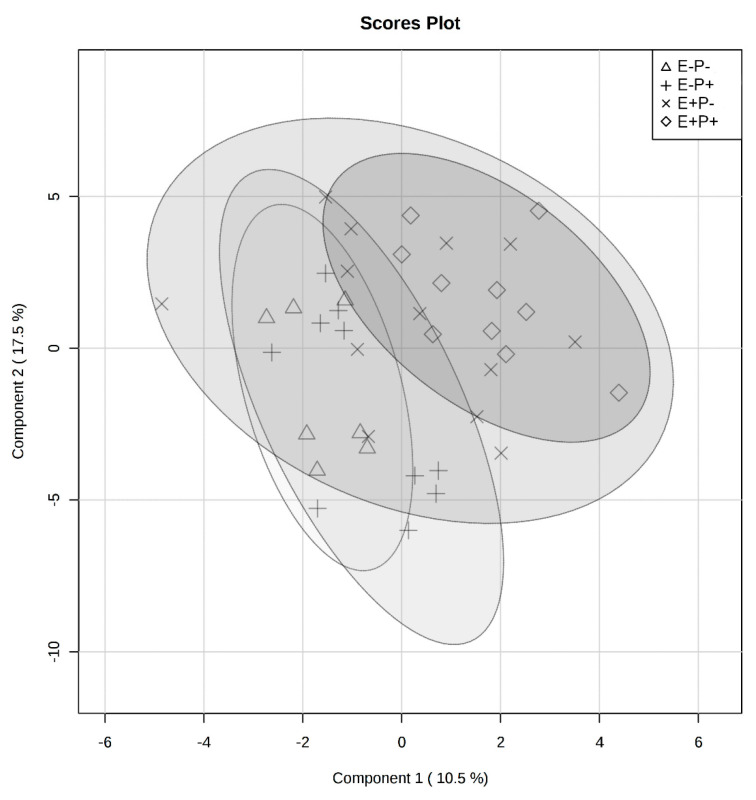
Partial least squares discriminant analysis (PLS-DA) visualizing differences in serum metabolomes between all treatment groups: endophyte-free seed without isoflavones (triangle), endophyte-free with isoflavones (plus-sign), endophyte-infected without isoflavones (multiplication-sign), and endophyte-infected with isoflavones (diamond). Ellipse represents a 95% confidence interval.

**Figure 8:**
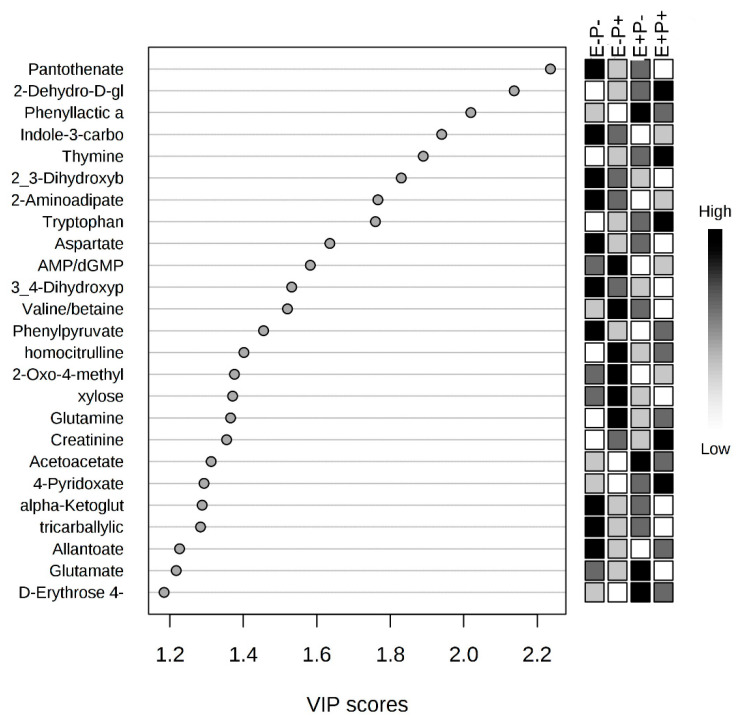
Variable importance in the projection (VIP) plot indicates pantothenate to have the greatest influence on the differences in serum metabolomes between all treatment groups.

All identified serum metabolites are presented in Supplementary File 2 with means and standard errors of the mean by treatment combination group. The serum metabolome was first analyzed by treatment combination group, isoflavone × seed type. The PLS-DA analysis indicated significant overlap among groups, with partial separation between the E+P+ and E−P− groups (Figure 7). Correlation analyses were performed to determine the correlation of individual serum metabolites with the treatment combination groups and VIP were generated to determine the metabolites that contributed to variation in serum metabolomes among treatment combination groups. Pantothenate was negatively correlated with interaction of seed type × isoflavone treatments (r = −0.29) and had the largest impact on metabolome differences, although not significant (*p* = 0.07; Figure 8).

**Figure 9:**
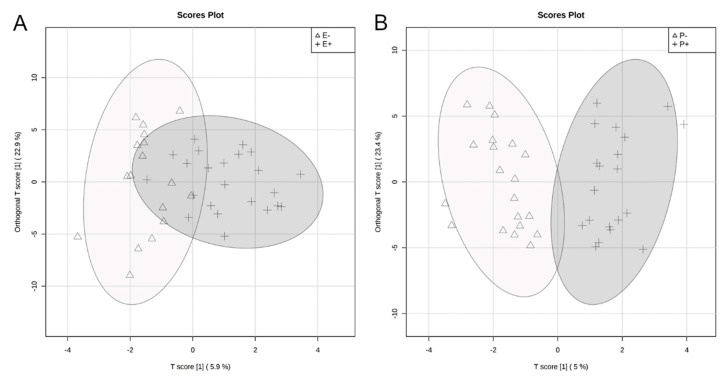
Orthogonal partial least squares discriminant analyses (O-PLS-DA) visualizing differences in serum metabolomes by seed type (**A**) and isoflavone (**B**) treatments. For seed type (**A**), endophyte-free (E−) steers are represented by a triangle and endophyte-infected (E+) steers by a plus-sign. For isoflavone treatments (**B**), steers receiving isoflavones (P+) are represented by a plus-sign and without isoflavones (P−) by a triangle. Ellipse represents a 95% confidence interval.

**Figure 10:**
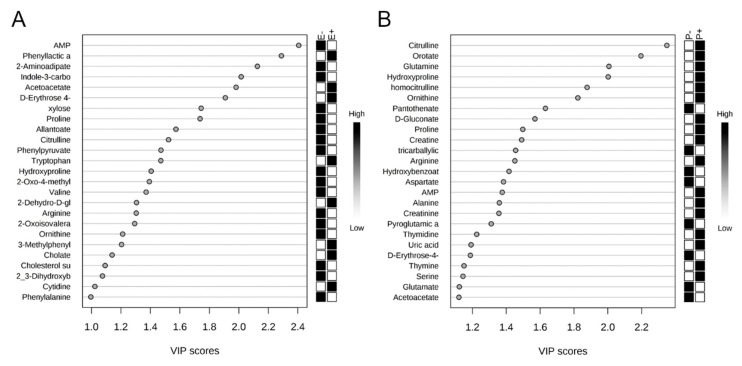
Variable importance in the projection (VIP) plot indicates AMP to have the greatest influence on the differences in serum metabolomes between endophyte-free (E−) and endophyte-infected (E+) seed treatment groups (**A**), and citrulline to have the greatest influence between isoflavone treated (P+) and control (P−) groups (**B**).

Similar to rumen fluid, no individual serum metabolites or metabolic pathways were affected by the interaction of seed type and isoflavone treatment (*p* > 0.05). The serum metabolome was then analyzed by the main effects of seed type or isoflavone treatment. In order to visualize the difference in serum metabolomes, O-PLS-DA analyses were generated for seed type (Figure 9A) and isoflavone treatment (Figure 9B). For seed type, partial separation was observed between E+ and E− seed groups (Figure 9A). However, complete separation of serum metabolomes was illustrated between steers receiving isoflavones and those that did not receive isoflavones (Figure 9B). Correlation analysis indicated AMP was negatively correlated with seed treatment (r = −0.35) and determined by VIP analysis to have the greatest impact on serum metabolome differences between E+ and E− steers (*p* = 0.03; Figure 10A). Between isoflavone treatment groups, citrulline was positively correlated (r = 0.47) and had the greatest impact on serum metabolome differences (*p* = 0.003; Figure 10B). Seven metabolites differed significantly as a result of isoflavone treatment (*p* < 0.05, Table 3), while no metabolites differed as a result of seed type (*p* > 0.05). Thirteen metabolic pathways differed (*p* < 0.05) as a result of seed type including glyoxylate and dicarboxylate metabolism; arginine biosynthesis; and alanine, aspartate, and glutamate metabolism (*p* < 0.01; Table 4). For isoflavone treatments, eight metabolic pathways were affected (*p* < 0.05), including pyrimidine metabolism and arginine and proline metabolism (*p* < 0.01; Table 4).

## 3. Discussion

The overall reductions in animal performance due to fescue toxicosis are estimated to cost the cattle industry over $2 billion annually [13,14]. Therefore, it is vital to discover management methods to reduce the impact and improve the efficiency of beef production. The objective of the current study was to use untargeted metabolomics to evaluate tall fescue seed and isoflavone consumption effects on metabolic intermediates, outputs, and pathways in the rumen and serum.

The metabolomes of the rumen and circulatory environments were first compared, independent of treatment groups, which observed distinctly unique metabolomes according to principal coordinate and abundance analyses. Highly abundant metabolites in each environment were not shared or only present in low abundances between the two environments. The metabolites identified between these different body systems are likely a result of the specific physiological functions of each system in the ruminant. The microbiome is a major contributor to rumen metabolome, as it supplies over 70% of the ruminant’s required nutrients [15]. These microbes are highly metabolically active in order to break down feedstuffs and release metabolites to complement host metabolism of which metabolites originate from the plants and other feedstuffs consumed [16]. Therefore, the majority of metabolites identified in the rumen are of xenobiotic origin. By evaluating the rumen metabolome, the effects of tall fescue seed and isoflavone consumption on rumen microbial metabolic processes can be inferred. The serum metabolites, however, are a result of absorbed metabolic products from the rumen and other organs. Tissues throughout the body produce intermediary metabolites from protein, carbohydrate, and lipid metabolism for energy production to perform physiological functions. These metabolites are then absorbed into the blood and can travel through the circulatory system to other tissues for further catabolic or anabolic processing. Therefore, the metabolome of the circulatory system is typically dominated by endogenous metabolites. Because of this systemic nature, metabolites in blood have been used as potential biomarkers to predict feed utilization [17] and production parameters [18], as well as evaluate responses to disease [19,20] and stress [21]. Evaluating the serum metabolome will determine the systemic metabolic response to tall fescue seed and isoflavone supplementation. Together, the effects on individual metabolites and metabolic pathways in rumen fluid and serum will determine how alterations in microbial and host metabolism contribute to symptoms of fescue toxicosis or the benefits isoflavones may contribute to mitigate these detrimental impacts.

Reductions in average daily gain and delayed development of beef cattle are a major consequence of fescue toxicosis [8,22]. The rumen microbiome is crucial for providing nutrients needed by the host for energy requirements and muscle development. As the microbiome has previously been shown to be affected by consuming endophyte infected tall fescue [12,23], the metabolites and other products produced by the rumen microorganisms may be altered, potentially contributing to reductions in growth and feed efficiency. The metabolites produced by the rumen microorganisms are a result of the richness of the rumen microbiome. Several of these metabolites released may be related to the consumption of ergot alkaloids concentrated on the endophyte infected tall fescue seed. Many of the rumen metabolites have a relationship with purine, carbohydrate, and nucleic acid metabolism, such as hypoxanthine, xylose, and uracil, respectively [24,25]; these metabolites are related to feed efficiency parameters. Clemmons et al. [26] found that these metabolites are bio-indicators of feed efficiency in cattle showing low residual feed intake. Interestingly, they are negatively correlated to seed type in the rumen fluid of the current study; it is evident that animals are being affected by the detrimental symptoms of tall fescue toxicosis, failing to gain weight, and being less feed-efficient. Additionally, we did not observe a large number of different metabolites because of the reduction of the rumen microbiome. As a normal rumen environment, the significant presence of other metabolites, which are crucial for the ergot alkaloids metabolism and production of volatile fatty acids, was expected and improves feed efficiency and rumen microbial richness.

Vasoconstriction, induced by ergot alkaloids, occurs throughout the body, resulting in multiple observed symptoms of fescue toxicosis [4,6]. Specifically, the contractility of the mesenteric vasculature surrounding the digestive tract is affected by the consumption of ergot alkaloids, potentially affecting nutrient absorption and subsequent host metabolism [5]. However, the consumption of isoflavones promotes vasodilation to increase blood flow and mitigate fescue toxicosis effects [10]. Ideally, oxygen and nutrient delivery to tissues is improved, thus benefiting host metabolism. Evaluating serum metabolites during induced fescue toxicosis and treatment with isoflavones may indicate the changes in metabolism systemically. The serum metabolome in the current study was greatly affected by isoflavone treatments with complete separation of animals’ global serum metabolomes between treatment groups; no metabolites differed as a result of seed type. Citrulline was identified as having the greatest influence on serum metabolomes between isoflavone treatment groups. Citrulline is an intermediary metabolite in the urea cycle, a metabolic process crucial for providing non-protein nitrogen to the ruminant [27]. As isoflavones inhibit hyper-ammonia producing bacteria in the rumen, this reduces the amount of protein degradation, leading to decreased ammonia and nitrogen availability [11,28]. Multiple metabolic pathways related to the urea cycle such as arginine biosynthesis and metabolism, pyrimidine metabolism, and nitrogen metabolism were affected in serum metabolites by isoflavone treatments. Therefore, the effect of citrulline on the serum metabolome due to isoflavone treatment may be a result of changes in the urea cycle by improving protein availability to the ruminant for muscle development.

Pantothenate was considered to be a major contributor to differences observed in the global serum metabolome among all treatment combinations, but was significantly higher in abundance in steers receiving isoflavone treatment. Previous studies have indicated different levels of pantothenate in the serum of animals differing in feed efficiency, with more feed efficient animals having greater serum levels of pantothenate [17,26]. Pantothenate is a key intermediary metabolite for the formation of Coenzyme A, which is crucial for amino acid and lipid metabolism for ruminant muscle development [29]. Additionally, the majority of metabolic pathways affected by isoflavone treatment in the current study were related to amino acid metabolism and biosynthesis. As animals experiencing fescue toxicosis often have low average daily gains [8], the use of isoflavone supplementation may mitigate the weight gain and growth consequences of fescue toxicosis. The greater amount of available pantothenate in the serum of animals consuming isoflavones, with changes in the urea cycling of the ruminant, may improve growth and muscle development in steers affected by fescue toxicosis.

A classic symptom of fescue toxicosis is a significant reduction of prolactin secretion; this is due to the similar homology of ergot alkaloids with the neurotransmitter dopamine. Ergot alkaloids will act as an agonist by binding dopamine receptors, preventing the release of prolactin [3,30]. Tyrosine is a precursor for the generation of the neurotransmitter dopamine [31]. The current study found tyrosine metabolism to be affected by seed type in the rumen; tyrosine metabolic pathways are influenced as the signals for dopamine production are reduced during fescue toxicosis. Additionally, the study found tryptophan metabolism was affected by seed type in the serum. Tryptophan is a key amino acid in regulating protein synthesis, specifically in muscle development, of multiple species [32,33]. The effects of tryptophan on muscle development are through the IGF-1 pathway [33]. Tryptophan is a precursor to the neurotransmitter serotonin, which stimulates the production of IGF-1 [34]. Supplementation of rumen protected tryptophan improved weight gain and feed efficiency of ruminants [35,36]. The observed impact of seed type on tryptophan metabolism in the serum may indicate reduced production of serotonin and subsequent IGF-1 signaling for muscle development. Together, the impacts of fescue toxicosis on tyrosine and tryptophan metabolism were also observed previously in the plasma of steers consuming endophyte-infected or endophyte-free seed [37,38]. Therefore, tall fescue seed consumption alters neurotransmitter development, leading to commonly observed symptoms of fescue toxicosis.

## 4. Conclusions

In conclusion, the rumen metabolome was largely impacted by seed type, while the serum metabolome was influenced by isoflavone supplementation. In the rumen, the impact of the seed type involved carbohydrate and nucleic acids metabolism products of the fescue seed diet inclusion. In the serum, differences in global metabolomes and individual metabolites involved in urea cycling and amino acid metabolic pathways were identified in animals receiving isoflavones and those that did not. Although the low dose of isoflavones administered to cattle indicated effects on the serum and rumen metabolome, further research is needed to determine the effects at other doses. Future applications may lead to the dietary inclusion of isoflavones to reduce the harmful effects of tall fescue toxicosis.

## 5. Materials and Methods

All experimental procedures involving animals were approved by the University of Tennessee Institutional Animal Care and Use Committee. The ethic approval code (IACUC) was 2540-0617 and was approved on 20 June 2017.

### 5.1. Experimental Design and Sample Collection

Experimental design, animal treatments, and sample collection methods have been previously described in Melchior et al. [12]. Briefly, this study used 36 purebred Angus steers of approximately eight months of age weighing 250 ± 20 kg from Ames Plantation in Grand Junction, TN. Steers were transported to the Plateau Research and Education Center (PREC) in Crossville, TN for the trial, as previously described [12]. Steers were allowed a 10 d acclimation period to the diet formulated to provide 11.57% crude protein and 76.93% total digestible nutrients (DM basis). The GrowSafe System© (GrowSafe Systems Ltd., Calgary, AB, Canada) was used to monitor feed intake. Prior to the beginning of the trial, steers were genotyped for the DRD2 receptor gene, which can influence cattle’s response to fescue toxicity [12]. Using this information, the study was blocked on DRD2 genotype, implementing a randomized complete block design. A 2 × 2 factorial arrangement of treatments was utilized with two types of tall fescue including endophyte-infected (E+) and endophyte-free fescue (E−), and treatment with Promensil© (P+) or without (P−) to provide red clover isoflavones. This combination of treatments resulted in four treatment groups: (1) endophyte-infected seed without Promensil (E+P−), (2) endophyte-infected with Promensil (E+P+), (3) endophyte-free without Promensil (E−P−), and (4) endophyte-free with Promensil (E−P+). Within each genotype block, steers were randomly assigned to treatments with nine steers per treatment group. The feed trial occurred over 21 days. In order to provide a consistent amount of ergot alkaloids, endophyte-infected tall fescue seed heads were incorporated into feed to provide a minimum of 0.011 mg ergovaline plus ergovalinine × kg of body weight^−1^ (BW) per day [3]. Seed heads were ground through a 5 mm screen using a Wiley Mill (Thomas Scientific, Swedesboro, NJ, USA) and included in feed. A total of 943 mg of isoflavones was provided daily before morning feeding based on previously established dosages [10] using a 28.4 g bolus (Torpac, Inc., Fairfield, NJ, USA) to provide 24.7 g of Promensil. Melchior et al. [12] previously reported the analysis and information of the components present in Promensil, and steers’ response to endophyte-infected seed and altered performance parameters. On the final day of the trial (day 21), approximately 9 mL of blood was collected from the coccygeal vein using a serum separator tube (Corvac, Sherwood Medical., St. Louis, MO, USA), and approximately 100 mL of rumen content was collected via oro-gastric lavage. Blood samples were centrifuged at 2000× *g* and 4 °C for 20 min, and serum was transferred to 2 mL microvials and stored at −80 °C until metabolite extractions. Rumen samples were centrifuged at 6000× *g* at 4 °C for 20 min. The supernatant was aspirated and filtered through a 0.22 µm syringe filter, transferred to 2 mL microvials, and stored at −80 °C until metabolite extraction.

### 5.2. Metabolite Extraction and Identification

Metabolites were extracted and analyzed as previously described [17] at the UTK Biological and Small Molecule Mass Spectrometry Core (BSMMSC). Briefly, 50 µL of filtered rumen fluid and 50 µL of serum from each steer were extracted using 0.1% formic acid in acetonitrile/water/methanol (2:2:1) using a previously described method [39]. Mobile phases consisted of A: 97:3 water/methanol with 11 mM tributylamine and 15 mM acetic acid and B: methanol, and a gradient consisting of the following: 0.0 min, 0% B; 2.5 min 0% B; 5.0 min, 20% B; 7.5 min, 20% B; 13 min, 55% B; 15.5 min, 95% B; 18.5 min, 95% B; 19 min, 0% B; and 25 min, 0% B; Synergy Hydro-RP column (100 × 2 mm, 2.5 μm particle size) was used to separate metabolites. The flow rate was set to a constant 200 μL/min and the column temperature was kept at 25 °C. A Dionex UltiMate 3000 UPLC system (Thermo Fisher Scientific, Waltham, MA) with an autosampler tray maintained at 4 °C was used to introduce a 10 µL sample to an Exactive Plus Orbitrap MS (Thermo Fisher Scientific, Waltham, MA, USA) using negative electrospray ionization (ESI) with a capillary temperature of 300 °C; spray voltage of 3 kV; and nitrogen sheath and sweep gas at 25 and 3 units, respectively. Data acquisition was done in negative ion mode with a full-scan covering the range of 72–1000 *m/z* at 140,000 resolution with automatic gain control of 3 × 10^6^ ions [40]. Metabolites are annotated using exact mass of the [M−H]− (±5 pmm) ion and known retention times (±0.3 min) generated from an in-house curated database. The database was created from the analysis of authentic standards and consisted of 300 compounds across various metabolic pathways, focusing on water soluble metabolites in pathways conserved among a diverse array of organisms.

### 5.3. Metabolite Identification

Data were analyzed similarly to those of Clemmons et al. [17]. The Xcalibur MS software (Thermo Electron Corp., Waltham, MA) was used to produce raw files, which were then converted to mzML format using ProteoWizard [41]. The software package Metabolomic Analysis and Visualization Engine for LC–MS Data (MAVEN) [42] was used to identify peaks using converted mzML files. MAVEN identifies metabolites based on non-linear retention time correction and calculates peak areas across samples, using a preliminary mass error of ±20 ppm and a retention time window of 5 min. The UTK BSMMSC used a library of 263 retention time-accurate *m/z* pairs taken from MS1 spectra for final metabolite annotations. These are based on expansions of previous work [40] and have been replicated at the UTK BSMMSC. The eluted peak of the annotated metabolite had to be found within 2 min of the expected retention time, and the metabolite mass had to be within ±5 ppm of the expected value to be identified as a known compound. The compound area of each peak was calculated using the Quan Browser function of the Xcalibur MS Software (Thermo Electron Corp., Waltham, MA, USA).

### 5.4. Data Analysis

Metabolomic data were analyzed using MetaboAnalyst 4.0 [43] and SAS 9.4 (SAS Institute, Cary, NC, USA). For data analysis in MetaboAnalyst, data were first pre-processed. Metabolite data were filtered using interquartile range, normalized by median, log transformed, and auto scaled prior to analysis in MetaboAnalyst 4.0. First, the rumen and serum metabolomes were collectively compared in order to determine similarity of rumen fluid and serum metabolomes for possible overlap or comparison. The rumen and serum metabolomes were visualized using orthogonal partial least squares discriminant analysis (O-PLS-DA) and partial least squares discriminant analysis (PLS-DA) with 2000 permutations. Model fitting for the O-PLS-DA was assessed using R2Y with prediction power determined using the Q2 metric. A heatmap was generated with the top 25 metabolites to illustrate differences in serum and rumen fluid metabolomes by steer. Next, rumen fluid and serum metabolomes were analyzed separately by treatment combination (i.e., isoflavone × seed type), isoflavone, and seed type. Within each of these, data were visualized via PCA and O-PLS-DA with 2000 permutations, and metabolomes by individual steers were illustrated using heatmaps. Correlation analyses between the top 25 metabolites and treatment groups or combinations were performed for both rumen fluid and serum metabolomes, as well as variable importance in projections (VIP) of the top 25 metabolites. Finally, pathway analyses were performed to determine metabolic pathways that were significantly impacted in rumen fluid and serum by isoflavone or seed type using a global test with relative-betweenness centrality and a reference pathway of Escherichia coli K-12 MG1655 [44].

Raw data were further analyzed in SAS 9.4 (SAS Institute, Cary, NC, USA), First, data were analyzed for normality using the UNIVARIATE procedure, and were considered normal with a Shapiro–Wilk statistic of ≥0.90 and visual observation of histograms and q-q plots. Data that were normally distributed were analyzed with a mixed model analysis of variance (ANOVA) using the GLIMMIX procedure with fixed effects of seed type, isoflavone treatment, and their interaction with the random effect of genotype × isoflavone × seed type. Metabolites that did not follow a normal distribution were fixed ranked and then analyzed using a mixed model ANOVA using the GLIMMIX procedure with fixed effects of seed type, isoflavone treatment, and their interaction with random effect of genotype × isoflavone × seed type.

## Figures and Tables

**Table 1:** Rumen fluid metabolites that significantly differed by seed type.

Metabolite	Seed Type ^†^		*p* Value ^€^
	E+	E−	
Dihydroxybenzoate	5.90 × 10^7^ ± 5.35 × 10^6 B^	8.43 × 10^7^ ± 6.26 × 10^6 A^	0.05
Adenine *	2.30 × 10^7^ ± 1.13 × 10^7 B^	6.84 × 10^7^ ± 1.32 × 10^7 A^	0.02
CMP *	9.17 × 10^5^ ± 7.64 × 10^5 B^	3.17 × 10^6^ ± 8.95 × 10^5 A^	0.04
Deoxyuridine *	8.04 × 10^5^ ± 2.71 × 10^5 B^	1.74 × 10^6^ ± 3.18 × 10^5 A^	0.02
Glutamate *	7.18 × 10^7^ ± 2.17 × 10^7 B^	1.57 × 10^8^ ± 2.54 × 10^7 A^	0.05
Guanosine *	3.00 × 10^5^ ± 1.44 × 10^5 B^	8.63 × 10^5^ ± 1.69 × 10^5 A^	0.05
Homoserine/threonine	1.02 × 10^7^ ± 8.90 × 10^5 B^	6.65 × 10^6^ ± 7.60 × 10^5 A^	0.05
Hypoxanthine *	4.40 × 10^7^ ± 1.66 × 10^7 B^	1.17 × 10^8^ ± 1.94 × 10^7 A^	0.01
Uracil *	5.76 × 10^7^ ± 1.19 × 10^7 B^	1.08 × 10^8^ ± 1.39 × 10^7 A^	0.02
Xanthine *	1.79 × 10^8^ ± 4.34 × 10^7 B^	3.48 × 10^8^ ± 5.09 × 10^7 A^	0.01
Xylose *	3.63 × 10^6^ ± 1.05 × 10^6 B^	8.69 × 10^6^ ± 1.23 × 10^6 A^	0.01

* Analysis based on ranked data; ^†^ values are measured as mean ± SEM of area under the peak; ^€^ significance determined at *p* ≤ 0.05 based on FDR-corrected *p*-values; ^AB^ within-row represent groupings based on Fisher’s LSD.

**Table 2:** Rumen fluid metabolic pathways impacted by seed type or isoflavone treatments.

Pathway	FDR	Impact	*p* Value
**Seed Type**			
Purine metabolism	2.73 × 10^−4^	0.338	6.89 × 10^−6^
Arginine and proline metabolism	2.73 × 10^−4^	0.075	1.07 × 10^−5^
Pentose and glucuronate interconversions	3.42 × 10^−4^	0	2.01 × 10^−5^
Beta-Alanine metabolism	3.69 × 10^−4^	0	2.89 × 10^−5^
Pyrimidine metabolism	3.99 × 10^−4^	0.494	5.92 × 10^−5^
Pantothenate and CoA biosynthesis	3.99 × 10^−4^	0.229	6.7 × 10^−5^
Aminoacyl-tRNA biosynthesis	3.99 × 10^−4^	0.2	7.81 × 10^−5^
Tyrosine metabolism	3.99 × 10^−4^	0	7.83 × 10^−5^
Novobiocin biosynthesis	3.99 × 10^−4^	0	7.83 × 10^−5^
Thiamine metabolism	3.99 × 10^−4^	0	7.83 × 10^−5^
Phenylalanine metabolism	7.94 × 10^−4^	0.001	1.71 × 10^−4^
Phenylalanine, tyrosine, and tryptophan biosynthesis	0.001	4.60 × 10^−4^	2.78 × 10^−4^
Carbapenem biosynthesis	0.001	0	3.25 × 10^−4^
Butanoate metabolism	0.001	0	3.25 × 10^−4^
Porphyrin and chlorophyll metabolism	0.001	0	3.25 × 10^−4^
Pentose phosphate pathway	0.003	0.07	0.001
Amino sugar and nucleotide sugar metabolism	0.004	0.109	0.001
Glutathione metabolism	0.004	0.014	0.002
D-Glutamine and D-glutamate metabolism	0.004	0.172	0.002
Nitrogen metabolism	0.004	0	0.002
**Isoflavone Treatment**			
Methane metabolism	0.84824	0.154	0.032
Sulfur metabolism	0.84824	0	0.033

**Table 3:** Individual serum metabolites that significantly differed by isoflavone treatment.

Metabolite	Isoflavone Treatment ^†^		*p* Value ^€^
	P+	P−	
Histidine *	8.50 × 10^6^ ± 8.94 × 10^5^	1.05 × 10^7^ ± 9.37 × 10^5^	0.05
Cytidine *	1.51 × 10^6^ ± 4.89 × 10^5 B^	2.67 × 10^7^ ± 5.12 × 10^5 A^	0.01
Pantothenate	6.64 × 10^6^ ± 1.84 × 10^6 B^	1.51 × 10^7^ ± 1.93 × 10^6 A^	0.01
Homocysteine	1.47 × 10^6^ ± 1.28 × 10^5 B^	2.02 × 10^6^ ± 1.35 × 10^5 A^	0.02
Allantoin	1.94 × 10^8^ ± 1.14 × 10^7 B^	2.37 × 10^8^ ± 1.19 × 10^7 A^	0.03
GABA	9.68 × 10^5^ ± 1.40 × 10^5 B^	1.41 × 10^6^ ± 1.44 × 10^5 A^	0.05
Methylhistidine	8.35 × 10^5^ ± 6.25 × 10^4^	1.04 × 10^6^ ± 6.51 × 10^4^	0.05

* Analysis based on ranked data; ^†^ values are measured as mean ± SEM of area under the peak; ^€^ significance determined at *p* ≤ 0.05 based on FDR-corrected *p*-values; ^AB^ within-row represent groupings based on Fisher’s LSD.

**Table 4:** Serum metabolic pathways affected by seed type and isoflavone treatments.

Pathway	FDR	Impact	*p* Value
**Seed Type**			
Glyoxylate and dicarboxylate metabolism	0.013	0.11	0.005
Arginine biosynthesis	0.013	0.51	0.006
Alanine, aspartate, and glutamate metabolism	0.015	0.73	0.007
Cysteine and methionine metabolism	0.051	0.14	0.024
Glycine, serine, and threonine metabolism	0.054	0.16	0.029
Ubiquinone and other terpenoid-quinone biosynthesis	0.054	0	0.029
Aminobenzoate degradation	0.054	0	0.029
Vitamin B6 metabolism	0.069	0.05	0.039
Monobactam biosynthesis	0.069	0	0.041
Lysine biosynthesis	0.069	0	0.041
Nicotinate and nicotinamide metabolism	0.069	0.06	0.042
Tryptophan metabolism	0.07	0	0.044
Cyanoamino acid metabolism	0.076	0	0.05
**Isoflavone Treatment**			
Pyrimidine metabolism	0.151	0.37	0.007
Arginine and proline metabolism	0.151	0.19	0.008
D-glutamine and D-glutamate metabolism	0.151	0.17	0.013
Nitrogen metabolism	0.151	0	0.013
Arginine biosynthesis	0.151	0.51	0.015
Glutathione metabolism	0.173	0.01	0.02
Purine metabolism	0.209	0.09	0.029
Glyoxylate and dicarboxylate metabolism	0.269	0.13	0.045

## Supplementary Materials

The following are available online at https://www.mdpi.com/2072-6651/12/12/744/s1, Supplementary File 1: Means and standard error of the means of all metabolites in the rumen fluid between treatment groups; Supplementary File 2: Means and standard error of the means of all metabolites in the serum between treatment groups.
